# Spatiotemporal dynamics and multiple driving factors of antimicrobial resistance in China during the COVID-19 pandemic (2019–2023): a provincial panel data analysis

**DOI:** 10.1128/aac.01600-25

**Published:** 2026-02-09

**Authors:** Xu Zheng, Xiaoyan You, Yu Liu, Binwei Wu

**Affiliations:** 1 Qingdao Municipal Hospital, Qingdao, China; 2 College of Geodesy and Geomatics, Shandong University of Science and Technology 727765, Qingdao, China; Shionogi Inc., Florham Park, New Jersey, USA

**Keywords:** antimicrobial resistance, one health, spatial autocorrelation, machine learning, driving factors, China

## Abstract

Antimicrobial resistance (AMR) poses a critical and growing global health threat, directly causing millions of deaths, with China bearing a significant burden. Understanding the provincial dynamics and multifactorial one health drivers of AMR, especially amidst the transformative 2019–2023 coronavirus disease 2019 (COVID-19) pandemic, remains crucial but underexplored. This comprehensive study investigated the spatiotemporal patterns and multisectoral drivers of methicillin-resistant *Staphylococcus aureus* (MRSA), carbapenem-resistant *Klebsiella pneumoniae* (CRKP), and carbapenem-resistant *Acinetobacter baumannii* (CRAB) prevalence across Chinese provinces using a robust 2019–2023 panel data set. Utilizing spatial autocorrelation (Global Moran’s I) and a multimodel approach, including panel fixed-effects regression, least absolute shrinkage and selection operator, and random forest, we identified robust drivers across healthcare, agricultural, environmental, and socioeconomic domains. Significant positive spatial autocorrelation was found for CRKP (Moran’s *I* = 0.225; *P* < 0.05) and CRAB (Moran’s *I* = 0.159; *P* < 0.05), indicating geographical clustering, whereas MRSA exhibited no significant pattern. Pathogen-specific drivers emerged. MRSA prevalence was linked to livestock inventory and PM2.5; CRKP to healthcare expenditure and pig inventory; and CRAB to healthcare expenditure and hospital beds, alongside counterintuitive negative associations with population aging and average length of hospital stay. The direct annual effect of COVID-19 was not statistically significant. We conclude that Chinese AMR is a spatially heterogeneous challenge driven by complex one health factors. A striking “paradox of progress” suggests higher healthcare capacity correlates with dangerously increased carbapenem-resistant pathogens, emphasizing the urgent need for robust infection prevention and control. The pandemic’s influence was predominantly indirect. These findings demand multisectoral, regionally tailored AMR strategies integrating healthcare, agricultural, and environmental policies for effective control.

## INTRODUCTION

Antimicrobial resistance (AMR) is a leading global health threat of the 21st century, undermining modern medicine and straining healthcare systems. In 2019 alone, bacterial AMR was the direct cause of 1.27 million deaths globally and was associated with an estimated 4.95 million deaths, in which a drug-resistant infection played a role (1). A substantial portion of this burden was concentrated in the Western Pacific region. As the world’s largest consumer and producer of antibiotics, China is central to global AMR containment efforts and faces substantial challenges within this high-burden region (2).

The emergence and spread of AMR are shaped by a complex interplay of clinical, agricultural, environmental, and socioeconomic factors under the one health framework (3). Key drivers include antibiotic overuse in human medicine and livestock production, which accelerates the selection and dissemination of resistant strains (4–6). Environmental pathways are increasingly recognized; for example, through the discharge of wastewater from hospitals and agricultural settings, which introduces antibiotic residues, resistant bacteria, and antibiotic resistance genes (ARGs) into aquatic and terrestrial ecosystems (7, 8). Moreover, air pollutants such as PM2.5 may act as carriers for ARGs, facilitating their regional spread (9). Healthcare system capacity and practices, including resource availability, infection prevention and control (IPC) effectiveness, and patient case mixing, also shape AMR epidemiology (10, 11). Socioeconomic conditions, population dynamics, and climate further modulate AMR through their effects on antibiotic use, sanitation, and pathogen transmission (12–14). However, many previous studies relied on cross-sectional data, potentially overlooking dynamic interactions and spatial dependencies, which can lead to biased estimates (15).

The COVID-19 pandemic further disrupted antibiotic use and AMR surveillance, imposing unique pressures on health systems. For example, the widespread empirical use of antibiotics (e.g., azithromycin) for potential co-infections, coupled with surges of critically ill patients requiring mechanical ventilation, led to intensified prescribing of broad-spectrum agents (16, 17). Simultaneously, IPC programs were often strained, and antimicrobial stewardship teams were frequently repurposed for pandemic response, potentially weakening AMR containment efforts (18). Consequently, the evidence suggests that complex and heterogeneous impacts of the pandemic on antibiotic consumption and resistance trends across various settings (19). As the world’s largest consumer and producer of antibiotics, China plays a pivotal role in global AMR containment but also faces significant challenges (2). Despite advancements in antimicrobial stewardship and surveillance, considerable provincial disparities persist in economic development, healthcare system capacity, and public health infrastructure. While national-level surveillance provides an overall picture of AMR trends, how these provincial heterogeneities shape the distinct spatiotemporal dynamics and multifactorial drivers of AMR remains a critical but underexplored knowledge gap. These disparities may lead to unique spatial patterns and drivers of AMR.

This study uses a spatiotemporal framework to analyze three priority pathogens of high clinical importance: methicillin-resistant *Staphylococcus aureus* (MRSA) and two “critical priority” carbapenem-resistant gram-negative bacteria, *Klebsiella pneumoniae* (CRKP) and *Acinetobacter baumannii* (CRAB) (20, 21). We aimed to (i) map the spatial distribution and clustering of resistance across Chinese provinces from 2019 to 2023, a period covering the COVID-19 pandemic, and (ii) identify key drivers from the healthcare, agricultural, environmental, and socioeconomic domains via a robust multimodel approach. This strategy, which integrates panel fixed effects (PLM), least absolute shrinkage and selection operator (LASSO), and random forest (RF) models, was chosen to enhance the robustness of our findings. Each model offers unique strengths—controlling for unobserved heterogeneity (PLM), selecting key predictors from a high-dimensional data set (LASSO), and capturing complex nonlinear relationships (RF). By triangulating results across these diverse analytical frameworks, we can identify drivers that are consistent and less dependent on specific model assumptions, thereby increasing confidence in our conclusions (22, 23). Our objective is to provide evidence for the development of targeted, regionally tailored AMR containment strategies.

## MATERIALS AND METHODS

### Data sources and collection

This study’s data included bacterial resistance rates, antibiotic usage, environmental factors, and socioeconomic and healthcare resources. Data were primarily collected from publicly available sources published by official national institutions in China, covering the period from 2019 to 2023. This timeframe was chosen to analyze AMR dynamics both before and during the COVID-19 pandemic. Provincial administrative regions in China (*n* = 31, mainland China) served as the basic analytical units.

Bacterial resistance rate data: Sourced from the China Antimicrobial Resistance Surveillance System (CARSS), which was established by the Chinese Center for Disease Control and Prevention. Please note that while the official website may have regional access restrictions, the full English name is provided to facilitate the retrieval of its public reports and publications. We collected annual resistance rate data for major pathogens, including MRSA, CRKP, and CRAB, across provinces.

Antibiotic usage data: Obtained from the National Health Commission (https://www.nhc.gov.cn/) and CARSS. The key indicators included antibiotic consumption intensity per 100 patient-days, antibiotic utilization rates for hospitalized patients, and utilization rates of restricted-use antibiotics.

Environmental factor data: Acquired from the China Meteorological Administration (annual average temperature and precipitation data for each province, https://www.cma.gov.cn/) and the China National Environmental Monitoring Center (annual average concentrations of air pollutants such as PM2.5 and O3, https://www.cnemc.cn/en/).

Socioeconomic and healthcare resource data: All other macroeconomic socioeconomic indicators (e.g., per capita GDP, per capita local government healthcare expenditure, per capita local government environmental protection expenditure, proportion of population aged 65 and above, daily urban wastewater treatment capacity), healthcare resource indicators (e.g., hospital beds per 10,000 population, health technical personnel per 10,000 population, ICU beds per 10,000 population, average length of hospital stay [ALOS], inpatient admission rate), livestock farming-related indicators (e.g., per capita pig inventory, per capita poultry egg production, per capita large livestock inventory), and transportation indicators (e.g., passenger volume rate, passenger turnover) were obtained from various issues of the China Statistical Yearbook (https://www.stats.gov.cn/sj/ndsj/). All predictor abbreviations are defined in Table 1.

**TABLE 1:** List of abbreviations and definitions

Abbreviation	Full definition
Antibiotic use	
ABU_Int	Intensity of antibiotic use
IP_AMU_Rate	Inpatient antimicrobial use rate
SGA_ABU_Rate	Special grade antibiotic use rate
Environmental factors	
Ann_Prec	Annual precipitation
Avg_O3	Annual average O3 concentration
Avg_PM25	Annual average PM2.5 concentration
Avg_Temp	Average temperature
PC_UG_Area	Per capita urban green area
Socioeconomics and public services	
PC_GDP	Per capita GDP
PC_LF_EnvExp	Per capita local fiscal environmental protection expenditure
PC_LF_MedExp	Per capita local fiscal medical and health expenditure
Pop65_Rate	Population aged 65 and over rate
UWW_Cap_10K	Urban wastewater treatment capacity per 10,000 population
Healthcare resources and services	
Avg_HospStay	Average hospital stay duration
HCW_10K	Healthcare workers per 10,000 population
HospBeds_10K	Hospital beds per 10,000 population
ICU_Beds_10K	ICU beds per 10,000 population
IP_Adm_Rate	Inpatient admission rate
Livestock farming	
PC_LrgLS_Own	Per capita large livestock ownership
PC_Pig_Own	Per capita pig ownership
PC_PoulEgg_Prod	Per capita poultry egg production
Population mobility	
PC_Pass_TMile	Per capita passenger turnover mileage
PC_Pass_Trips	Per capita passenger trips

### Data preprocessing

The annual bacterial resistance rates and potential influencing factors for each province were integrated into a panel data set. A one-period lag was created for the main antibiotic usage indicators. Bacterial resistance rates were Logit-transformed, and all predictor variables were standardized. Before model analysis, strict collinearity detection was performed on independent variables, including removing zero-variance variables and checking the rank of the model design matrix to ensure model identifiability.

### Spatial autocorrelation analysis

To assess the spatial clustering patterns of the bacterial resistance rates, spatial autocorrelation analysis was employed. A spatial weight matrix was constructed on the basis of the Queen contiguity criterion. Global Moran’s I was calculated to evaluate overall spatial association, and local indicators of spatial association (LISA) were used to identify high-high, low-low, high-low, and low-high spatial cluster types, which were then visualized through LISA cluster maps.

### Panel data regression models

To identify the driving factors of bacterial resistance and control for unobserved provincial and temporal heterogeneity, three panel data analysis methods were utilized:

PLM: We employed a two-way fixed effects model, which simultaneously incorporated individual fixed effects (for each province) and time fixed effects (annual dummy variables). This approach accounts for unobserved heterogeneity that might influence AMR outcomes, including inherent provincial characteristics and macrolevel annual shocks (e.g., the widespread impact of the COVID-19 pandemic). Significance testing for all coefficients utilized Huber–White heteroskedasticity-consistent robust standard errors to ensure the validity of inference in the presence of potential heteroskedasticity.

LASSO regression: Given the relatively high dimensionality and potential multicollinearity of our predictor variable set, we implemented LASSO regression. This regularization technique performs both variable selection and coefficient shrinkage simultaneously, effectively handling multicollinearity and identifying the most influential predictors. The optimal regularization parameter (lambda) was determined through 10-fold cross-validation. Predictors with nonzero coefficients were considered important.

RF: To capture nonlinear relationships and complex interactions between variables, an RF model was adopted. Before modeling, the dependent and independent variables were residualized by performing linear regression on province and year fixed effects, indirectly controlling for these fixed effects. Variable importance was assessed by the percentage increase in the mean squared error (%IncMSE).

The final key drivers were identified by summarizing the variables commonly identified across the three models (PLM significance, LASSO nonzero coefficients, and top 8 in RF importance).

The detailed outputs from the LASSO regression and RF models are available from the corresponding author upon reasonable request.

### Software tools

All data processing, statistical analysis, and plotting were performed in the R language (R-4.5.1) environment. The key R packages included dplyr, sf, spdep, leaflet, ggplot2, plm, glmnet, and randomForest.

## RESULTS

### Descriptive statistics of the variables

Descriptive statistics for all the dependent and predictor variables are presented in Table 2, which presents their means, standard deviations, minimums, and maximum values during the study period.

**TABLE 2:** Descriptive statistics of antimicrobial resistance and potential driving factors across Chinese provinces (2019−2023)^*a*^

Variable name	Unit	Mean	SD	Min	Max
Dependent variables (prevalence/resistance rate, %)					
MRSA prevalence rate	%	28.57	7.84	15.20	48.50
CRKP prevalence rate	%	9.50	7.01	0.20	32.80
CRAB prevalence rate	%	52.34	10.55	18.20	78.60
Antibiotic use					
Intensity of antibiotic use	DDD/100 bed-days	34.90	1.86	29.16	40.28
Inpatient antimicrobial use rate	%	43.97	2.88	36.84	51.71
Special grade antibiotic use rate	%	4.86	0.86	2.41	6.56
Socioeconomics and public services					
Per capita GDP	10,000 RMB/person	7.85	3.48	3.47	20.03
Population aged 65 and over rate	%	13.59	3.24	5.81	21.06
Per capita local fiscal medical and health expenditure	RMB/person	1,644.03	775.41	852.33	5,285.90
Per capita local fiscal environmental protection expenditure	RMB/person	524.32	317.25	140.10	1,837.21
Urban wastewater treatment capacity per 10,000 population	m³/day/10,000 population	2,310.70	707.86	1,247.38	4,595.51
Healthcare resources and services					
Hospital beds per 10,000 population	Beds/10,000 population	67.00	9.69	43.71	89.26
Inpatient admission rate	%	17.60	4.21	8.19	27.82
Average hospital stay duration	Days	9.15	0.94	7.70	17.20
Healthcare workers per 10,000 population	Persons/10,000 population	102.89	15.37	74.62	177.95
ICU beds per 10,000 population	Beds/10,000 population	0.47	0.13	0.20	0.88
Environmental factors					
Average temperature	°C	13.85	5.70	1.58	25.46
Annual precipitation	mm	964.83	506.10	141.30	2,114.30
Annual average PM2.5 concentration	µg/m³	32.35	9.88	8.90	59.87
Annual average O3 concentration	µg/m³	63.02	7.85	40.96	81.65
Per capita urban green area	m²/person	37.89	13.84	19.50	78.06
Livestock farming					
Per capita large livestock ownership	Heads/person	0.18	0.37	0.00	2.01
Per capita pig ownership	Heads/person	0.27	0.16	0.01	0.71
Per capita poultry egg production	kg/person	21.00	17.61	1.04	78.00
Population Mobility					
Per capita passenger turnover mileage	km/person	1,111.76	490.07	307.50	2,578.98
Per capita passenger trips	Trips/person	8.07	5.11	1.13	34.61

^*a*^ MRSA, methicillin-resistant *Staphylococcus aureus*; CRKP, carbapenem-resistant *Klebsiella pneumoniae*; CRAB, carbapenem-resistant *Acinetobacter baumannii*.

During the study period, the average provincial prevalence of MRSA, CRKP, and CRAB was 28.57%, 9.50%, and 52.34%, respectively. Notably, there was substantial variation across provinces, with CRKP rates ranging from a mere 0.20% to as high as 32.80%. Similar heterogeneity was observed in potential drivers, such as per capita GDP and healthcare expenditure, highlighting the diverse socioeconomic and healthcare landscapes across China.

### Spatial distribution of bacterial resistance rates

**Fig 1:**
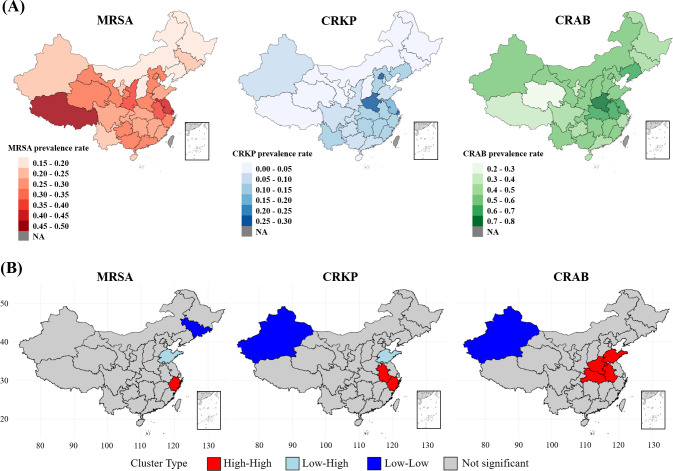
Spatial distribution and clustering of resistance rates for MRSA, CRKP, and CRAB. (**A**) Provincial average resistance rates (2019–2023). (**B**) LISA cluster maps identifying significant spatial hotspots (high-high) and cold spots (low-low). Nonsignificant provinces are shown in gray. MRSA, methicillin-resistant *Staphylococcus aureus*; CRKP, carbapenem-resistant *Klebsiella pneumoniae*; CRAB, carbapenem-resistant *Acinetobacter baumannii*. Base map is from the standard map service website of the ministry of natural resources of the people’s Republic of China; map approval no. GS (2019) 1822.

The national heatmaps illustrate the spatial distributions of the MRSA, CRKP, and CRAB prevalence rates from 2019 to 2023 (Fig. 1A). Global Moran’s *I* statistics revealed varied spatial patterns. Significant positive spatial autocorrelation was found for CRKP (Moran’s *I* = 0.225, *Z* = 2.269, *P* = 0.012) and CRAB (Moran’s *I* = 0.159, *Z* = 1.690, *P* = 0.045), indicating clear geographical clustering. In contrast, MRSA prevalence showed no significant spatial autocorrelation (Moran’s *I* = 0.109, *Z* = 1.222, *P* = 0.111), suggesting a more random spatial distribution. LISA analysis revealed specific hot spots (high-high) and cold spots (low-low) related to bacterial resistance rates (Fig. 1B).

### Analysis of the driving factors of bacterial resistance

**Fig 2:**
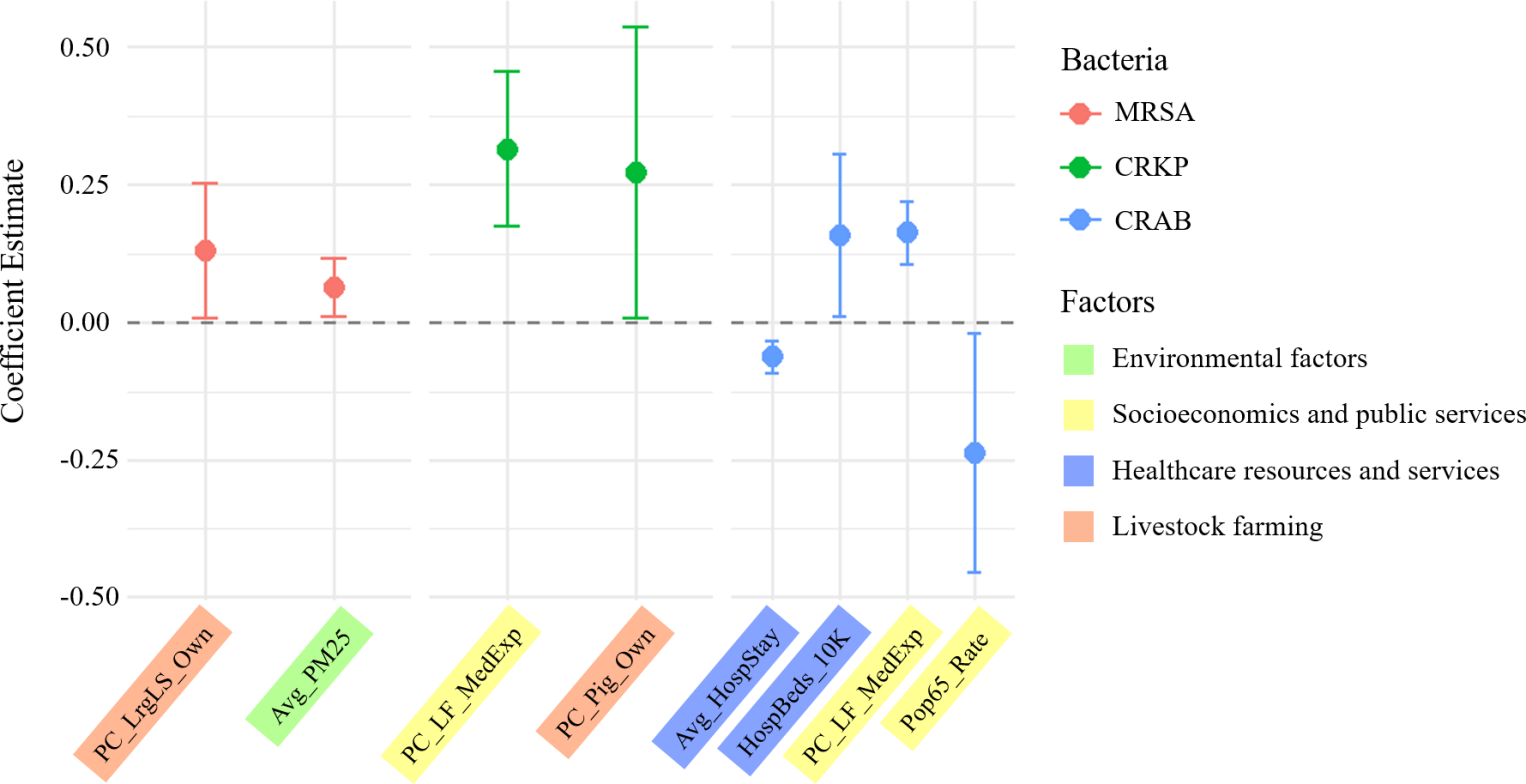
PLM estimates and 95% confidence intervals for factors associated with MRSA, CRKP, and CRAB prevalence rates in Chinese provinces. MRSA, methicillin-resistant *Staphylococcus aureus*; CRKP, carbapenem-resistant *Klebsiella pneumoniae*; CRAB, carbapenem-resistant *Acinetobacter baumannii*. All predictor abbreviations are defined in Table 1.

**Fig 3:**
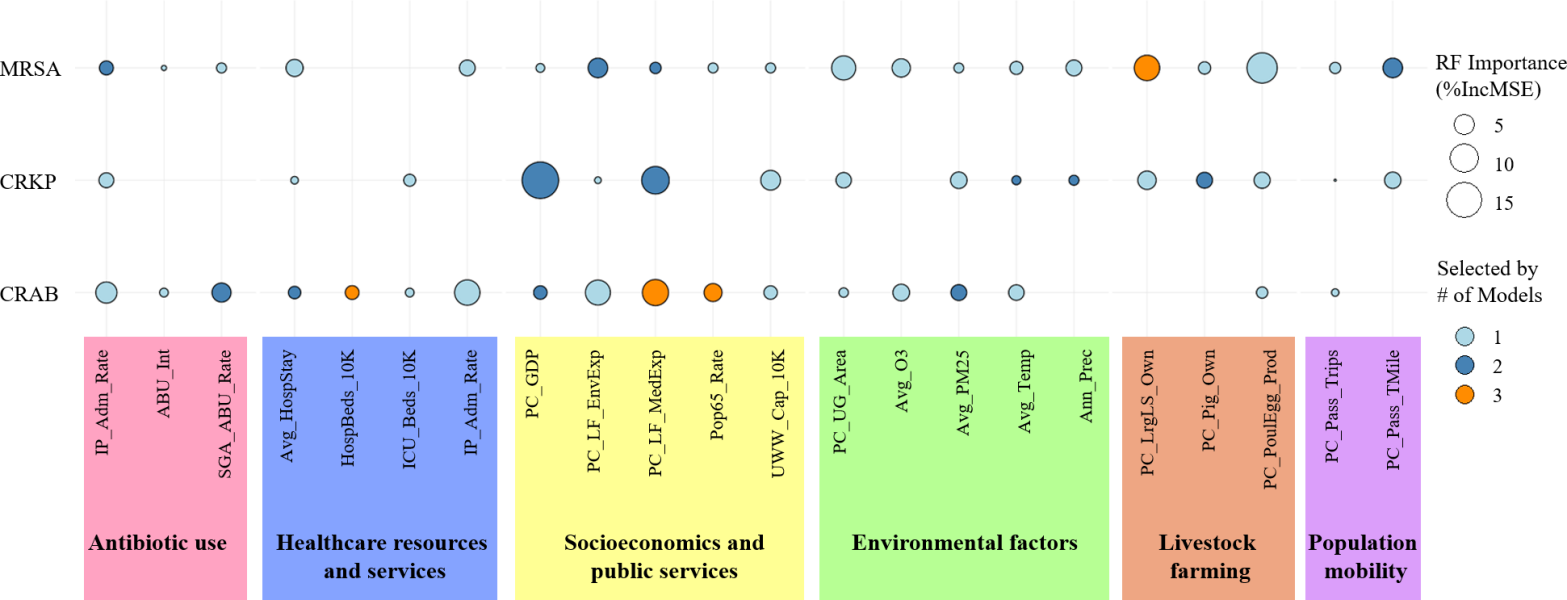
Multimodel synthesis of key drivers of MRSA, CRKP, and CRAB prevalence. The size of each bubble represents the variable’s importance from the random forest model (%IncMSE). The color intensity indicates model consensus—the number of models (out of three: PLM, LASSO, and RF) that identified the factor as significant. MRSA, methicillin-resistant *Staphylococcus aureus*; CRKP, carbapenem-resistant *Klebsiella pneumoniae*; CRAB, carbapenem-resistant *Acinetobacter baumannii*. All predictor abbreviations are defined in Table 1.

The combined results from the PLM, LASSO, and RF models identified pathogen-specific drivers for MRSA, CRKP, and CRAB (Fig. 2 and 3; Tables 3 and 4).

**TABLE 3:** Detailed panel fixed effects model results for factors associated with MRSA, CRKP, and CRAB prevalence rates^*a*^

Pathogen	Variable name	Estimate	Std. error	*t*-value	*P*-value
MRSA	Antibiotic use				
	Intensity of antibiotic use (lag 1)	−0.0187	0.0559	−0.3339	0.7392
	Inpatient antimicrobial use rate (lag 1)	0.0525	0.0346	1.5144	0.1332
	Special grade antibiotic use rate (lag 1)	−0.0168	0.0229	−0.7326	0.4656
	Environmental factors				
	Average temperature	−0.0297	0.0977	−0.3044	0.7614
	Annual precipitation	0.0031	0.0254	0.1203	0.9045
	Annual average PM2.5 concentration	0.0648	0.0270	2.3957	0.0185*
	Annual average O3 concentration	0.0050	0.0273	0.1816	0.8562
	Per capita urban green area	0.0622	0.0392	1.5850	0.1162
	Socioeconomics and public services				
	Urban wastewater treatment capacity per 10,000 population	−0.0349	0.0591	−0.5898	0.5567
	Population aged 65 and over rate	0.0117	0.0569	0.2050	0.8380
	Per capita GDP	−0.0006	0.0817	−0.0072	0.9942
	Per capita local fiscal medical and health expenditure	0.0042	0.0422	0.0987	0.9216
	Per capita local fiscal environmental protection expenditure	−0.0209	0.0139	−1.5044	0.1357
	Healthcare resources and services				
	Hospital beds per 10,000 population	0.0156	0.0398	0.3935	0.6948
	Healthcare workers per 10,000 population	−0.0894	0.0484	−1.8460	0.0679
	ICU beds per 10,000 population	0.0095	0.0240	0.3955	0.6933
	Average hospital stay duration	0.0016	0.0182	0.0858	0.9318
	Inpatient admission rate	0.0050	0.0298	0.1677	0.8672
	Livestock farming				
	Per capita pig ownership	−0.0219	0.0406	−0.5391	0.5910
	Per capita poultry egg production	−0.1292	0.0800	−1.6160	0.1094
	Per capita large livestock ownership	0.1312	0.0624	2.1016	0.0382*
	Population mobility				
	Per capita passenger trips	0.0150	0.0133	1.1278	0.2622
	Per capita passenger turnover mileage	0.0211	0.0247	0.8536	0.3954
	Control variables				
	Year 2020 (vs. 2019)	0.0614	0.0531	1.1547	0.2510
	Year 2021 (vs. 2019)	0.0879	0.0527	1.6665	0.0988
	Year 2022 (vs. 2019)	0.0825	0.0856	0.9634	0.3378
	Year 2023 (vs. 2019)	0.0687	0.0994	0.6913	0.4911
CRKP	Antibiotic use				
	Intensity of antibiotic use (lag 1)	−0.0540	0.1701	−0.3176	0.7515
	Inpatient antimicrobial use rate (lag 1)	0.0817	0.1430	0.5713	0.5691
	Special grade antibiotic use rate (lag 1)	0.0199	0.0764	0.2607	0.7949
	Environmental factors				
	Average temperature	−0.7446	0.4122	−1.8065	0.0739
	Annual precipitation	0.0223	0.0836	0.2670	0.7900
	Annual average PM2.5 concentration	−0.0058	0.1027	−0.0566	0.9550
	Annual average O3 concentration	0.1705	0.1494	1.1414	0.2565
	Per capita urban green area	0.1670	0.2257	0.7396	0.4613
	Socioeconomics and public services				
	Urban wastewater treatment capacity per 10,000 population	0.1667	0.1909	0.8732	0.3847
	Population aged 65 and over rate	−0.2933	0.2544	−1.1528	0.2518
	Per capita GDP	−0.5008	0.3184	−1.5727	0.1190
	Per capita local fiscal medical and health expenditure	0.3155	0.0719	4.3862	2.93E-05***
	Per capita local fiscal environmental protection expenditure	−0.2163	0.1179	−1.8341	0.0697
	Healthcare resources and services				
	Hospital beds per 10,000 population	0.0621	0.1417	0.4384	0.6620
	Healthcare workers per 10,000 population	−0.2281	0.2111	−1.0805	0.2826
	ICU beds per 10,000 population	−0.0057	0.1457	−0.0393	0.9687
	Average hospital stay duration	−0.0805	0.0406	−1.9824	0.0503
	Inpatient admission rate	0.0558	0.1716	0.3252	0.7458
	Livestock farming				
	Per capita pig ownership	0.2727	0.1347	2.0255	0.0456*
	Per capita poultry egg production	−0.0257	0.3405	−0.0755	0.9400
	Per capita large livestock ownership	0.9897	0.8467	1.1690	0.2453
	Population mobility				
	Per capita passenger trips	0.0588	0.0447	1.3164	0.1912
	Per capita passenger turnover mileage	−0.1400	0.1354	−1.0340	0.3037
	Control variables				
	Year 2020 (vs 2019)	−0.2932	0.1991	−1.4728	0.1440
	Year 2021 (vs 2019)	−0.0286	0.2678	−0.1067	0.9152
	Year 2022 (vs 2019)	−0.3019	0.3674	−0.8217	0.4133
	Year 2023 (vs 2019)	0.0684	0.3670	0.1863	0.8526
CRAB	Antibiotic use				
	Intensity of antibiotic use (lag 1)	0.0406	0.0627	0.6478	0.5187
	Inpatient antimicrobial use rate (lag 1)	0.0276	0.0627	0.4400	0.6609
	Special grade antibiotic use rate (lag 1)	−0.0425	0.0299	−1.4209	0.1585
	Environmental factors				
	Average temperature	0.0413	0.2330	0.1771	0.8598
	Annual precipitation	0.0339	0.0333	1.0167	0.3118
	Annual average PM2.5 concentration	0.0763	0.0533	1.4308	0.1557
	Annual average O3 concentration	0.0389	0.0559	0.6957	0.4883
	Per capita urban green area	−0.0501	0.0602	−0.8332	0.4068
	Socioeconomics and public services				
	Urban wastewater treatment capacity per 10,000 population	0.0822	0.0790	1.0404	0.3008
	Population aged 65 and over rate	−0.2364	0.1109	−2.1313	0.0356*
	Per capita GDP	−0.1118	0.1166	−0.9586	0.3402
	Per capita local fiscal medical and health expenditure	0.1635	0.0289	5.6488	1.61E-07***
	Per capita local fiscal environmental protection expenditure	−0.0371	0.0426	−0.8708	0.3860
	Healthcare resources and services				
	Hospital beds per 10,000 population	0.1590	0.0749	2.1237	0.0362*
	Healthcare workers per 10,000 population	0.0394	0.1135	0.3474	0.7291
	ICU beds per 10,000 population	0.0042	0.0598	0.0701	0.9443
	Average hospital stay duration	−0.0629	0.0151	−4.1596	6.89E-05***
	Inpatient admission rate	−0.1290	0.0677	−1.9065	0.0596
	Livestock farming				
	Per capita pig ownership	−0.0874	0.0706	−1.2385	0.2185
	Per capita poultry egg production	0.0383	0.1225	0.3128	0.7551
	Per capita large livestock ownership	0.0130	0.5844	0.0223	0.9823
	Population mobility				
	Per capita passenger trips	0.0263	0.0307	0.8579	0.3930
	Per capita passenger turnover mileage	0.0243	0.0483	0.5039	0.6155
	Control variables				
	Year 2020 (vs 2019)	−0.0666	0.0838	−0.7951	0.4285
	Year 2021 (vs 2019)	0.0634	0.1293	0.4903	0.6251
	Year 2022 (vs 2019)	−0.0135	0.1800	−0.0749	0.9404
	Year 2023 (vs 2019)	0.0776	0.2000	0.3879	0.6989

^*a*^ *P < 0.05, *** *P *< 0.001. MRSA, methicillin-resistant *Staphylococcus aureus*; CRKP, carbapenem-resistant *Klebsiella pneumoniae*; CRAB, carbapenem-resistant *Acinetobacter baumannii*.

**TABLE 4:** Multimodel analysis of factors associated with MRSA, CRKP, and CRAB prevalence rates in Chinese provinces^*a*^

Pathogen	Variable name	Panel fixed effects model (coefficient and *P*-value)	LASSO regression (selected)	Random forest (importance rank and %IncMSE)
MRSA	Antibiotic use			
	Intensity of antibiotic use (lag 1)	−0.019 (0.739)	TRUE	Not in top 8
	Inpatient antimicrobial use rate (lag 1)	0.052 (0.133)	TRUE	6 (2.128)
	Special grade antibiotic use rate (lag 1)	−0.017 (0.466)	TRUE	Not in top 8
	Environmental factors			
	Average temperature	−0.030 (0.761)	TRUE	Not in top 8
	Annual precipitation	0.003 (0.904)	TRUE	Not in top 8
	Annual average PM2.5 concentration	0.065 (0.0185)*	FALSE	Not in top 8
	Annual average O3 concentration	0.005 (0.856)	TRUE	Not in top 8
	Per capita urban green area	0.062 (0.116)	FALSE	3 (6.699)
	Socioeconomics and public services			
	Urban wastewater treatment capacity per 10,000 population	−0.035 (0.557)	TRUE	Not in top 8
	Population aged 65 and over rate	0.012 (0.838)	TRUE	Not in top 8
	Per capita GDP	−0.001 (0.994)	TRUE	Not in top 8
	Per capita local fiscal medical and health expenditure	0.004 (0.922)	TRUE	7 (1.346)
	Per capita local fiscal environmental protection expenditure	−0.021 (0.136)	TRUE	4 (4.342)
	Healthcare Resources and Services			
	Average hospital stay duration	0.002 (0.932)	TRUE	Not in top 8
	Inpatient admission rate	0.005 (0.867)	TRUE	Not in top 8
	Livestock farming			
	Per capita pig ownership	−0.022 (0.591)	TRUE	Not in top 8
	Per capita poultry egg production	−0.129 (0.109)	FALSE	1 (10.755)
	Per capita large livestock ownership	0.131 (0.0382)*	TRUE	2 (7.498)
	Population mobility			
	Per capita passenger trips	0.015 (0.262)	FALSE	8 (1.304)
	Per capita passenger turnover mileage	0.021 (0.395)	TRUE	5 (4.340)
CRKP	Environmental Factors			
	Average temperature	−0.745 (0.0739)	TRUE	7 (0.803)
	Annual precipitation	0.022 (0.790)	TRUE	6 (1.027)
	Annual average PM2.5 concentration	−0.006 (0.955)	TRUE	Not in top 8
	Per capita urban green area	0.167 (0.461)	FALSE	5 (2.610)
	Socioeconomics and public services			
	Urban wastewater treatment capacity per 10,000 population	0.167 (0.385)	FALSE	3 (4.412)
	Per capita GDP	−0.501 (0.119)	TRUE	1 (15.808)
	Per capita local fiscal medical and health expenditure	0.315 (2.93E-05)***	FALSE	2 (8.850)
	Per capita local fiscal environmentalprotectionn expenditure	−0.216 (0.0697)	TRUE	Not in top 8
	Healthcare resources and services			
	Average hospital stay duration	−0.081 (0.0503)	TRUE	Not in top 8
	Inpatient antimicrobial use rate (lag 1)	0.082 (0.569)	TRUE	Not in top 8
	ICU beds per 10,000 population	−0.006 (0.969)	TRUE	Not in top 8
	Livestock farming			
	Per capita pig ownership	0.273 (0.0456)*	TRUE	Not in top 8
	Per capita poultry egg production	−0.026 (0.940)	TRUE	Not in top 8
	Per capita large livestock ownership	0.990 (0.245)	TRUE	Not in top 8
	Population mobility			
	Per capita passenger trips	0.059 (0.191)	FALSE	8 (0.020)
	Per capita passenger turnover mileage	−0.140 (0.304)	FALSE	4 (2.986)
CRAB	Antibiotic use			
	Intensity of antibiotic use (lag 1)	0.041 (0.519)	TRUE	Not in top 8
	Inpatient antimicrobial use rate (lag 1)	0.028 (0.661)	FALSE	3 (5.130)
	Special grade antibiotic use rate (lag 1)	−0.042 (0.159)	TRUE	4 (4.113)
	Environmental factors			
	Average temperature	0.041 (0.860)	TRUE	Not in top 8
	Annual average PM2.5 concentration	0.076 (0.156)	TRUE	6 (2.786)
	Annual average O3 concentration	0.039 (0.488)	TRUE	Not in top 8
	Per capita urban green area	−0.050 (0.407)	TRUE	Not in top 8
	Socioeconomics and public services			
	Urban wastewater treatment capacity per 10,000 population	0.082 (0.301)	TRUE	Not in top 8
	Population aged 65 and over rate	−0.236 (0.0356)*	TRUE	5 (3.630)
	Per capita GDP	−0.112 (0.340)	TRUE	8 (1.971)
	Per capita local fiscal medical and health expenditure	0.163 (1.61E-07)***	TRUE	1 (7.935)
	Per capita local fiscal environmental protection expenditure	−0.037 (0.386)	TRUE	Not in top 8
	Healthcare resources and services			
	Hospital beds per 10,000 population	0.159 (0.0362)*	TRUE	7 (2.076)
	Average hospital stay duration	−0.063 (6.89E-05)***	TRUE	Not in top 8
	ICU beds per 10,000 population	0.004 (0.944)	TRUE	Not in top 8
	Inpatient admission rate	−0.129 (0.0596)	FALSE	2 (7.429)
	Livestock farming			
	Per capita poultry egg production	0.038 (0.755)	TRUE	Not in top 8
	Population mobility			
	Per capita passenger trips	0.026 (0.393)	TRUE	Not in top 8

^*a*^ *P < 0.05, *** *P* < 0.001. MRSA, methicillin-resistant *Staphylococcus aureus*; CRKP, carbapenem-resistant *Klebsiella pneumoniae*; CRAB, carbapenem-resistant *Acinetobacter baumannii*. All predictor abbreviations are defined in Table 1.

Drivers of MRSA prevalence rates: In the panel fixed effects model, PM2.5 (estimate = 0.065, *P* < 0.05) and per capita large livestock inventory (estimate = 0.131, *P* < 0.05) had significant positive effects on MRSA prevalence rates (Fig. 2; Table 3). LASSO regression identified multiple variables with nonzero coefficients, including lagged antibiotic use indicators, socioeconomic factors, environmental factors, healthcare resources, and livestock-related factors (Table 4). The RF model’s importance ranking indicated that per capita poultry egg output, per capita large livestock inventory, and per capita urban greenery area were the three most important predictors (Table 4). Collectively, livestock-related indicators and certain environmental factors play significant roles in MRSA prevalence (Fig. 3; Table 4).

Drivers of CRKP prevalence rates: The results from the panel fixed effects model revealed that per capita local medical and health expenditures (estimate=0.315, *P* < 0.001) had a significant positive effect on CRKP prevalence rates, and the per capita pig inventory (estimate=0.273, *P* < 0.05) also exhibited a significant positive association (Fig. 2; Table 3). The LASSO model selects multiple variables, including per capita GDP, PM2.5, average temperature, and some antibiotic use indicators (Table 4). The random forest model ranked per capita GDP, per capita local fiscal medical and health expenditures, and per capita urban sewage daily treatment capacity as the three most important variables (Table 4). These findings indicate a close relationship between healthcare investment, livestock farming scale, and socioeconomic development level and CRKP prevalence rates (Fig. 3; Table 4).

Drivers of CRAB prevalence rates: For CRAB prevalence rates, the panel fixed effects model identified several significant driving factors: per capita local medical and health expenditures (estimate = 0.163, *P* < 0.001) and hospital beds per 10,000 people (estimate = 0.159, *P* < 0.05) were significantly positively associated; conversely, the proportion of the population aged 65 and above (estimate = −0.236, *P* < 0.05) and ALOS (estimate = −0.063, *P* < 0.001) were significantly negatively correlated (Fig. 2; Table 3). Both the LASSO and RF models also extensively selected variables related to healthcare resources, demographic structure (e.g., proportion of the population aged 65+), and antibiotic use (e.g., lagged hospitalized patient antibiotic utilization rates) as important driving factors (Fig. 3; Table 4).

### Overall impact of the COVID-19 pandemic period

In the PLM analysis for all three pathogens, the year fixed effects (2020, 2021, 2022) were not statistically significant after controlling for other covariates (Table 3). This suggests that the direct annual effect of pandemic years was not statistically significant and that its impact was likely mediated through the other included covariates, such as changes in healthcare expenditure and patient mobility.

## DISCUSSION

Our spatiotemporal analysis of MRSA, CRKP, and CRAB in China from 2019 to 2023 revealed that AMR is a spatially heterogeneous challenge deeply embedded within a one health context. We found significant geographical clustering for CRKP and CRAB, but not MRSA, and identified pathogen-specific drivers spanning healthcare, agricultural, and environmental domains. Crucially, our findings highlight a “paradox of progress,” where higher healthcare capacity can be associated with greater resistance and reveal complex ecological associations that challenge individual-level risk assumptions.

The observed spatial clustering of CRKP and CRAB aligns with existing evidence on the interregional dissemination of high-risk clones, underscoring the interconnectedness of healthcare systems (24, 25). In contrast, the more dispersed pattern of MRSA, which showed no significant global spatial autocorrelation, likely reflects its dual epidemiology in both healthcare and community settings (26). This lack of autocorrelation explains a key finding from our spatial analysis (Fig. 1): while some individual provinces may exhibit high prevalence rates (appearing as “hot spots” on the prevalence map), they do not form statistically significant spatial clusters. A spatial cluster requires a high-prevalence province to be surrounded by other high-prevalence neighbors. The absence of such clusters for MRSA suggests that its drivers are either highly localized within provincial borders or are so broadly distributed that they do not create contiguous high-risk zones at the scale of our analysis. This finding stands in contrast to CRKP and CRAB, where significant clustering points towards strong inter-regional transmission dynamics. This pathogen-specific spatial dynamic underscores the need for tailored surveillance strategies.

Our multimodel approach consistently identified the agricultural sector as a key driver, particularly for MRSA and CRKP. The association with livestock inventories (large livestock, pigs) and poultry production highlights the critical role of antibiotic use in animal husbandry in shaping the human AMR landscape (27, 28). Furthermore, the link between PM2.5 and MRSA prevalence supports the hypothesis that air pollution can act as a vehicle for resistant bacteria and genes, contributing to their regional spread (9, 29). Interestingly, the random forest model also highlighted urban green space as an important predictor for MRSA, suggesting that ecological factors promoting microbial diversity might have a protective effect, a hypothesis worthy of further investigation (30).

For CRKP and CRAB, the positive association with provincial healthcare expenditure and hospital bed density exemplifies a “paradox of progress” (31). While expanded healthcare access is beneficial, it can inadvertently intensify nosocomial transmission and selective pressure if not matched by robust IPC and stewardship programs (10, 32). This was particularly relevant during the COVID-19 pandemic, where surges in patient volume often strained IPC capacity, leading to documented outbreaks of multidrug-resistant organisms (MDROs), such as CRKP and CRAB (33–35). Our machine learning models complemented these findings by identifying other crucial infrastructure and clinical practice elements. The high importance of urban sewage treatment capacity for CRKP (in RF) points to environmental sanitation as a critical control point, whereas the importance of inpatient antibiotic utilization for CRAB (in RF) directly confirms that clinical antibiotic pressure is a fundamental driver at the macroecological level (11, 36–38).

Notably, our study revealed two counterintuitive negative associations for CRAB prevalence that expose the complexities of ecological analysis. First, provinces with a greater proportion of the elderly population presented a lower prevalence of CRAB. This finding, while contrary to individual-level risk, may reflect macrolevel structural differences in healthcare delivery. Regions with older populations might have healthcare systems more oriented towards primary and chronic care than high-intensity tertiary hospitals, where CRAB transmission is most concentrated (39, 40). Aging populations could also serve as a proxy for a lower concentration of high-end medical facilities, thereby reducing carbapenem exposure (41, 42). Second, a longer ALOS was associated with a lower CRAB prevalence. This paradox can be interpreted in two nonmutually exclusive ways: (i) at the system level, a shorter ALOS could be a symptom of hospital overload and rapid patient turnover, which compromises IPC and accelerates cross-transmission (43); (ii) alternatively, in high-resistance settings, a shorter ALOS might be a deliberate clinical or administrative strategy to minimize patient exposure (44). These findings powerfully illustrate the potential for ecological fallacy and underscore that macrolevel indicators often reflect complex system dynamics rather than simple aggregations of individual risks.

The absence of a statistically significant direct effect for the pandemic years (2020–2022) does not imply a lack of impact. Rather, it suggests that the pandemic’s influence was largely mediated through the covariates in our model, such as fluctuations in healthcare expenditure, patient mobility, and prescribing patterns, which our analysis controlled for.

Several limitations should be acknowledged. First, our study utilizes data from the CARSS). Despite its extensive coverage of over 6,900 institutions in 31 provinces, the CARSS employs a passive surveillance model. This approach may lead to an over-representation of data from large, well-resourced tertiary hospitals, which possess more robust laboratory and reporting capacities than their smaller counterparts. This potential selection bias could influence the observed resistance rates and their associations, warranting caution when interpreting the findings. Second, as an ecological study, it cannot establish individual-level causality, and the potential for ecological fallacy, as discussed, is inherent. Furthermore, while we included a wide range of predictors, data on several potentially important factors were unavailable at the provincial level for the study period. These include granular data on the consumption of specific antibiotic classes (e.g., carbapenems), quantitative metrics of IPC adherence, and genomic surveillance data, which would be invaluable for tracking the spread of specific high-risk clones. The inclusion of such data in future work could provide deeper insights and make the analysis more robust. Finally, while year fixed effects were used, more specific pandemic-related indicators (e.g., lockdown intensity) could offer deeper insights into their indirect effects in future work.

This spatiotemporal analysis revealed that in China, the drivers of AMR are pathogen specific, spatially clustered, and deeply rooted in a one health framework. We provide evidence for a “paradox of progress” in healthcare development and highlight complex ecological associations that challenge simplistic interpretations of AMR drivers. The impact of the COVID-19 pandemic appears to be indirect and is mediated through systemic pressures. Our findings underscore the urgent need for multisectoral, regionally tailored strategies that integrate antimicrobial stewardship, IPC, agricultural antibiotic regulation, and environmental management to effectively contain AMR.

Looking forward, a practical one health approach in China could involve establishing provincial-level interdepartmental task forces, comprising public health, agricultural, and environmental protection agencies, to coordinate integrated surveillance and interventions, a framework consistent with global recommendations (45). For instance, based on our finding linking livestock to AMR, policies could focus on restricting the use of critically important antimicrobials in animal husbandry, a strategy whose effectiveness in reducing AMR has been demonstrated in multiple settings (46). Similarly, the association between healthcare capacity and carbapenem resistance highlights an urgent need to mandate and audit robust IPC programs as a prerequisite for hospital expansion or accreditation. Future research should also focus on pilot testing these integrated interventions at a regional level to evaluate their effectiveness and cost-efficiency, providing a scalable model for national AMR control.

## Data Availability

The national surveillance data on bacterial resistance and antibiotic usage were provided by the China Antimicrobial Resistance Surveillance System (CARSS). These official datasets are subject to restrictions as their official data portal has regional network access limitations, which prevents direct public hyperlink access. Therefore, the data supporting this study’s findings are available from the authors upon reasonable request and with the permission of CARSS.
